# Comparing a mindfulness- and CBT-based guided self-help Internet- and mobile-based intervention against a waiting list control condition as treatment for adults with frequent cannabis use: a randomized controlled trial of CANreduce 3.0

**DOI:** 10.1186/s12888-022-03802-9

**Published:** 2022-03-24

**Authors:** Michelle Dey, Andreas Wenger, Christian Baumgartner, Ute Herrmann, Mareike Augsburger, Severin Haug, Doris Malischnig, Michael P. Schaub

**Affiliations:** 1 Swiss Research Institute for Public Health and Addiction at the University of Zurich, Konradstrasse 32, 8005 Zurich, Switzerland; 2 Cantonal Hospital Winterthur, Institute for Anesthesiology, Pain Center, Brauerstrasse 15, 8401 Winterthur, Switzerland; 3 Office of Addiction and Drug Policy of Vienna, Institute for Addiction Prevention, Modecenterstrasse 14, 1030 Vienna, Austria

**Keywords:** Cannabis, Internet-based intervention, Self-help, Mindfulness, Cognitive-behavioral therapy, Randomized controlled trial

## Abstract

**Background:**

Though Internet- and mobile-based interventions (IMIs) and mindfulness-based interventions (generally delivered *in-situ*) appear effective for people with substance use disorders, IMIs incorporating mindfulness are largely missing, including those targeting frequent cannabis use.

**Methods:**

This paper details the protocol for a three-arm randomized controlled trial comparing a mindfulness-based self-help IMI (arm 1) and cognitive-behavioral therapy (CBT)-based self-help IMI (arm 2) versus being on a waiting list (arm 3) in their effectiveness reducing cannabis use in frequent cannabis users. Predictors of retention, adherence and treatment outcomes will be identified and similarities between the two active intervention arms explored. Both active interventions last six weeks and consist of eight modules designed to reduce cannabis use and common mental health symptoms. With a targeted sample size of *n* = 210 per treatment arm, data will be collected at baseline immediately before program use is initiated; at six weeks, immediately after program completion; and at three and six months post baseline assessment to assess the retention of any gains achieved during treatment.

The primary outcome will be number of days of cannabis use over the preceding 30 days. Secondary outcomes will include further measures of cannabis use and use of other substances, changes in mental health symptoms and mindfulness, client satisfaction, intervention retention and adherence, and adverse effects. Data analysis will follow ITT principles and primarily employ (generalized) linear mixed models.

**Discussion:**

This RCT will provide important insights into the effectiveness of an IMI integrating mindfulness to reduce cannabis use in frequent cannabis users.

**Trial registration:**

International Standard Randomized Controlled Trial Number Registry: ISRCTN14971662; date of registration: 09/09/2021.

## Background

The consumption of cannabis to a tetrahydrocannabinol (THC) level of 1% or greater is generally prohibited in Switzerland [1]. Nevertheless, it is – as in other countries [2, 3] – one of the most commonly used illicit substances, with about a third of individuals at least 15-years-old having used cannabis at least once [4]. Lifetime prevalence is highest among young adults (especially males), with more than half of 20–34-year-olds reporting they have used cannabis at least once [4]. In this age group, roughly 10% indicate that they have used cannabis within the previous 30 days, with almost 25% of these admitting (almost) daily use [4]. People who use cannabis have a one in five risk of developing a cannabis use disorder (CUD), and this risk increases if cannabis use is initiated early and it is used at least weekly [5]. People with a CUD are more likely to report symptoms of other mental disorders (e.g., mood disorders [6]) and frequent cannabis use is associated with negative physical and social consequences [7].

However, even people with CUD who perceive a need for treatment might be reluctant to seek traditional professional help. Reasons include their fear of stigmatization, a preference to rely on themselves, and financial and structural barriers [8, 9]. Self-help internet- and mobile-based interventions (IMI) help overcome these barriers. According to meta-analyses, Internet- and computer-based interventions seem to be effective at reducing cannabis use in the short-term, with small but significant effect sizes (ES) observed post-treatment [10, 11] and significant effects at 3-month [12] and 6-month [13], but not 12-month follow-up [10]. The studies that were included in the aforementioned meta-analyses predominantly used personalized feedback, motivational interviewing (MI) and/or cognitive-behavioral therapeutic (CBT) approaches.

CANreduce is also a self-help IMI for frequent cannabis users that draws on classical MI [14] and CBT approaches [15]. The program consists of eight modules that users can work through at their own pace over a period of six weeks. Two randomized three-armed controlled trials (RCT) have been conducted to test the effectiveness of versions of the program in existence at the time. Version 1.0 [16] was shown to be more effective reducing cannabis use when supplemented with the option of a brief professional chat (counseling) session, even though this option only was used by about one fifth of users. More precisely, small ES (0.20) were identified for the comparison between the study arm with chat counseling versus a waiting list control group at 3-month follow-up [17]. Hence, the chat invitation that was sent out by a health professional was possibly already sufficient to reduce participants’ cannabis use.

Based on this finding and following the theory behind the supportive-accountability model [18], it was hypothesized that a more automated eCoach would – similar to the health professional used in CANreduce 1.0 – increase both the program’s rate of adherence and effectiveness [19]. The eCoach who was incorporated in the first study arm of CANreduce 2.0 introduced the contents of most modules via a short video. Furthermore, weekly semiautomatic motivational and adherence-focused guidance-based feedback was sent out in her name, as were answers to any questions raised by users (in actuality, the questions were answered by anyone on the study team). The second study arm of CANreduce 2.0 only differed from the first arm by referring to an anonymous support team rather than to a personal eCoach (i.e., the same semiautomatic emails were sent to participants and all questions participants raised were answered in the same way), albeit without any videos. The second study arm, which had the anonymous support team, was found to be the most effective intervention, in terms of reducing cannabis use, with a moderate ES (d = 0.60) versus waiting list controls at 3-month follow-up [20]. Moreover, in this second study arm, CUD symptoms (measured using Cannabis Use Disorders Identification Test-Revised (CUDIT-R) [21], d = 0.52), cannabis dependence (Severity of Dependence Scale (SDS) [22], d = 0.60) and anxiety symptoms (d = 0.51) were reduced, this last reduction documented for the first time testing an IMI for cannabis users. That the ES for cannabis use reduction were much larger than in CANreduce 1.0 (see above) might also have been due to further adaptions that were implemented in both active study arms in CANreduce 2.0. Most importantly, frequently co-occurring mental health problems (e.g., symptoms of depression and anxiety, and sleep difficulties) among frequent cannabis users were addressed in CANreduce 2.0 via CBT-based approaches for depression and anxiety reduction and via social problem-solving skills training [23–25]. This adaptation was crucial, since comorbidities between frequent cannabis use and other mental disorders might dampen a treatment’s effectiveness [26]. Despite the larger ES observed with CANreduce 2.0, compared to both the previous version of the program and earlier digital interventions (ES = 0.11 [12]), there remains room for improvement.

One potential way to further improve the program would be to incorporate previously-neglected but promising approaches within IMI, including mindfulness. *Mindfulness* can be defined as the “awareness that emerges through paying attention on purpose, in the present moment and nonjudgmentally to the unfolding of experience moment by moment” [27]. Practicing mindfulness can be useful to people with substance use disorders (SUD), since their condition may be characterized by *mindlessness* [28] (i.e., habitual or stereotyped responses that may be carried out automatically without conscious volition or strategic consideration of the consequences that might arise from them [29]). Mindfulness has been suggested as a way to support people with SUD via various mechanisms [29, 30]; e.g., by helping them increase their awareness of triggers, habitual patterns, and ‘automatic’ reactions and by shifting their relationship to all internal and external experiences [31]. Mindfulness practices — like ‘mindful breathing’ — might help individuals to reorient their attention to the sensation of breathing when they are experiencing something distressing that typically would trigger them to use a particular substance [31]. There also is evidence that mindfulness techniques (e.g., mindful breathing) can evoke biological (e.g., altered brain activity) and subsequently behavioral mechanisms (e.g., decreased substance cue-reactivity) that may lead to improved clinical outcomes (e.g., decreased craving) [29].

Though some standardized mindfulness-based interventions (MBI) were not originally developed specifically for people with SUD (e.g., Mindfulness-Based Stress Reduction (MBSR; [32]), they are nevertheless sometimes applied to this target group [33]. Other interventions — like Mindfulness-Based Relapse Prevention (MBRP; [31]), Mindfulness Training for Smokers (MTS; [34–36]) and Mindfulness-Oriented Recovery Enhancement (MORE; [37]) — have been conceptualized specifically for people with a SUD. MBI – including those previously mentioned – are mostly conducted in an in situ group therapy format over several weeks (typically 8 weeks). During group sessions, clients are guided by a trained professional through various mindfulness practices that are subsequently debriefed in group processes that is typically followed-up by an introduction to new psychoeducational material [29]. They also are given homework, including mindfulness practices and assignments to self-monitor symptoms related to their SUD [29].

Several papers have addressed the question of whether the above-mentioned standardized and other MBI are effective treating people with SUD. In one systematic review and meta-analysis published by Li et al. [38], MBI were found to have a small effect reducing substance misuse, a medium effect reducing cravings, and a large effect reducing levels of stress relative to alternative treatments (e.g., treatment as usual (TAU), CBT, and a support group). Similarly, Cavicchioli et al. [39] detected small to large effects for MBI relative to other active programs for alcohol and drug use disorders (small effects for abstinence, levels of perceived stress, and avoidance coping strategies; moderate effects for symptoms of anxiety and depression; large effects for level of perceived craving, negative affectivity, and post-traumatic symptoms). Other meta-analyses and systematic reviews have also yielded promising results concerning the effectiveness of MBI treating SUD [33, 40–44]. One systematic review and meta-analysis that specifically focused on MBRP for SUD concluded that this MBI might – relative to other interventions – exert small effects on withdrawal/cravings and the negative consequences of substance use, but no effects on other outcomes (including relapse, frequency of use, symptoms of anxiety or depression) [45]. In summary, at least some published evidence exists that MBI might be helpful to people with SUD. Furthermore, MBI might be superior to other treatments for some subgroups with certain clinical (e.g., co-occurring SUD and depression [46, 47]) or sociodemographic characteristics (e.g. [48]).

Despite these promising findings, that most studies on the effectiveness of MBI hitherto focused on substances like tobacco, alcohol, or poly-substance use [33] must be considered. To the best of our knowledge, the effectiveness of MBIs treating frequent cannabis use has, to date, only been evaluated in two pilot studies. In the first, Dakwar and Levin [49] provided 10 weeks of weekly, individual mindfulness-based psychotherapy to 14 cannabis-dependent subjects. Of these, 11 completed the program (79%), and eight achieved abstinence (57% by intent-to-treat (ITT) analysis) by the end of treatment. In a second study, by de Dios et al. [50], the efficacy of a brief, two-sessions intervention that combined MI and mindfulness meditation was examined as a means to reduce cannabis use in young adult females. The intervention group consisted of 22 women, while an assessment-only control group numbered 12. Women randomized to the intervention group used cannabis on fewer days than controls one, two, and three months post treatment. Thus, even though these two pilot studies are limited by their small size, they nonetheless both suggest that MBI might also be feasible and effective for frequent cannabis users.

The need for larger, well-designed RCTs to study the effectiveness of MBI is not specific to frequent cannabis user, but other substance-related problems, as well [30]. Furthermore, Wilson et al. [30] have provided suggestions on how future research could address the implementation challenges of traditional MBIs. Among other things, these authors pointed out the lack of studies evaluating MBI delivered via technology-based platforms, even though this mode of delivery could expand such interventions’ reach [30].

Preliminary findings on web- and/or mobile-based MBI for SUD are promising [30]. However, to the best of our knowledge, no paper on the effectiveness or efficacy of mindfulness-based web self-help for frequent cannabis users has yet been published. This said, Hides et al. [51] have published their protocol for an RCT to determine the efficacy and cost-effectiveness of a web-based program that includes a small mindfulness component compared to an information-only control website among young cannabis users (16–25 years) with psychotic experiences (i.e., a narrow target group).

Based on the outlined research on the effectiveness and efficacy of web-based self-help interventions, as well as MBI, for people with SUD, and based upon existing research gaps (e.g., the lack of large RCTs and any web-based MBI for frequent cannabis users), the study presented in this protocol seeks to examine the effectiveness of a web self-help intervention (CANreduce 3.0) that incorporates mindfulness as a way to reduce cannabis use among adults, of any age, who use cannabis frequently.

## Study design and methods

### Design

The self-help IMI called *CANreduce 3.0* will be evaluated within a three-arm RCT, which will compare a mindfulness-based self-help IMI (arm 1); a CBT-based self-help IMI (arm 2); and a waiting-list control condition (arm 3) in their effectiveness reducing cannabis use among frequent cannabis users. Since the mindfulness-based self-help IMI (arm 1) contains newly-developed contents, testing its effectiveness is of primary interest in the current RCT. The CBT-based self-help IMI (arm 2) is a slightly-adapted version of CANreduce 2.0 [19] and serves as a reference program [52]. Performance of the reference intervention is, thus, not the main consideration; however, if the test (arm 1) and reference intervention (arm 2) both fail to demonstrate a statistically-significant advantage over the control condition, this could suggest that the trial is insensitive, or lacks assay sensitivity. If differences are observed, the difference between the reference intervention (arm 2) and controls can be used to help assess the practical relevance of the difference between arm 1 and the control condition [52].

Consistent with this, a confidence interval approach will be used as an exploratory tool to identify similarities between treatment arms 1 and 2 [53, 54]. The control condition (arm 3) will consist of all the same assessments administered to the other two subject groups (see Section Measures), as well as brief advice on the health risks of cannabis use (e.g., potential short-term and long-term effects). Some screening and brief internet interventions from Swiss addiction counselling also are available on the Internet and, as such, might be accessed by controls, but no links to them will be provided in CANreduce 3.0. Participants who are assigned to the control group will be offered the opportunity to receive the intervention delivered in study arm 1 after they complete the final assessment of the RCT, six months after they register for the study).

The trial has been registered with the ISRCTN registry (date of registration: 09/09/2021) and is traceable as ISRCTN14971662 (http://www.isrctn.com/ISRCTN14971662). It also has been approved by the Ethics Committee of the Faculty of Arts and Social Science at the University of Zurich, Switzerland (approval number: 20.4.17). Any important protocol modifications would be reported to this Ethics Committee.

### Trial flow

**Fig. 1:**
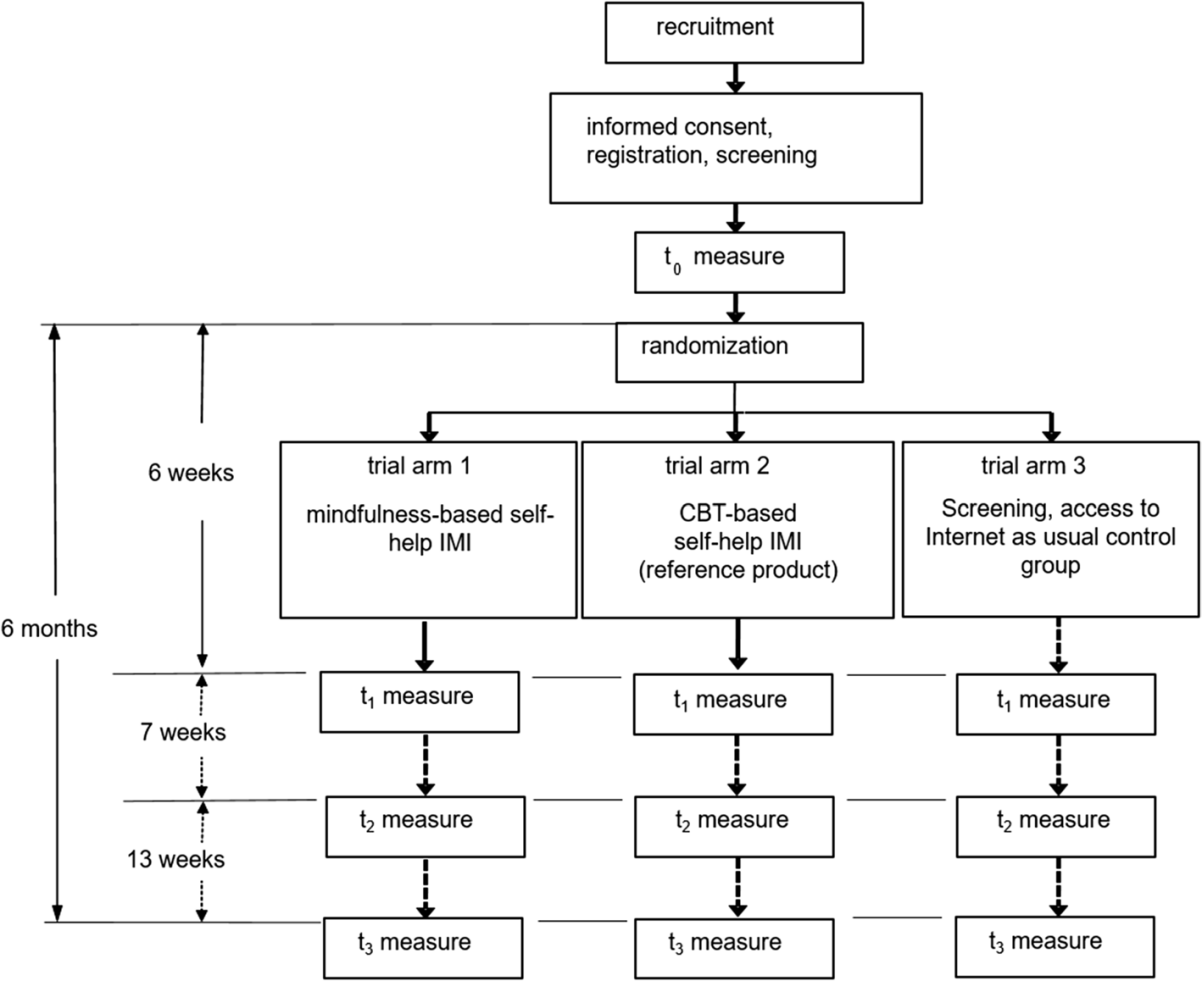
Trial flowchart

All of the steps in this RCT, including subject recruitment, the consent procedure, eligibility screening, the baseline and further assessments, and the randomization of participants to one of the three study arms are depicted in Fig. 1 and described in greater detail in subsequent sections.

#### Recruitment of study participants

Frequent cannabis users from the general population will be recruited directly through the website *canreduce.ch*, which is well-known since earlier versions of the program have been advertised broadly [19]. The target group also will be informed about the study via reports in community newspapers and posts on the national addiction internet-portal SafeZone.ch. Additionally, relevant healthcare professionals will be informed about the program via journals published by certain professional association to which they might belong (e.g., Swiss Medical Association FMH, Federation of Swiss Psychologists FSP) and related websites (e.g., praxissuchtmedizin.ch), as well as through direct mailings (including flyers to general practitioners, psychotherapists, and psychologists).

The recruitment phase, which has already started, began on September 13 2021 and will last until September 13 2023. The two-year duration of the recruitment phase should allow us to recruit the targeted sample size of 630 participants (see below). As detailed below, participants with sufficient knowledge of German are eligible to participate. Hence, even though the program is primarily advertised in the German-speaking part of Switzerland, it is possible that people from other parts of Switzerland, and from Germany and Austria will participate as well.

#### Consent procedure and registration

People interested in the program will first read a detailed description of the study, which will include all of the following elements:

- background and purpose of the study
- eligibility criteria for participation (see below)
- randomized allocation to one of three study arms (including a one in three chance of being assigned to a waiting-list control group)
- description of what individuals in each study arm are offered (no details about the differences between the two active study arms are provided, but they are broadly described as a revised version of the effective self-help IMI *CANreduce 2.0*)
- data collection points (baseline, t1, t2, t3)
- compensation for completing the follow-up questionnaires (see below)
- voluntariness of participation and their right to withdraw consent at any time, with no consequences
- confidentiality and data protection (anonymity is ensured by deleting all contact details of participants before statistical analysis and data archiving)
- advantages of study participation (participation might help them reduce their cannabis use; that the program is free also is mentioned)
- potential risks of study participation (that CANreduce cannot replace a professional diagnosis and/or face-to-face therapy is emphasized; that symptoms of withdrawal might occur when cannabis use is reduced initially is mentioned)
- contacts to health professionals (under which circumstances professional help should be sought is described); emergency contacts and a search index to find health professionals can be retrieved from the CANreduce-website
- institutional framework of the study, responsible project leader and contact details for queries
- prior approval of the study by the Ethics Committee of the Faculty of Arts and Social Science at the University of Zurich (including contact details for any complaints about the study).

Once informed about the study, potential participants must give informed consent by confirming that (1) they have read and understood the study information; (2) they are currently not receiving any other treatment to reduce their cannabis use; (3) they are participating voluntarily and understand that they may terminate their participation at any time, without giving any reasons; and (4) by agreeing to participate, they are permitting their data — anonymized and aggregated with data from others collected over the course of the study — can be both analyzed and made available to others within an open repository. Once individuals provide such consent, they will be allowed to register for the study by providing a username, and both an email address and a phone number with which they can be contacted.

#### Screening for eligibility

After providing informed consent and registering for the study, potential participants are screened for eligibility. Inclusion and exclusion criteria are listed in Table 1.

**Table 1:** Inclusion/exclusion criteria and their underlying rationale

**Inclusion Criteria**	**Rationale**
1) Informed consent	To ensure participants’ knowledge of what the study entails and to meet the requirements of the ethics committee
2) Minimal age of 18 years	To ensure that all participants are of legal age to consent on their own
3) Cannabis use at least once weekly over the last 30 days	To include participants not only with (almost) daily use and, thus, increase the study’s validity and generalizability
4) At least once weekly Internet access, including the potential to listen to audio(-visual) files while undisturbed	To ensure at least some access to the intervention and – in study arm 2 – to the mindfulness practices and video material
4) A valid email address and telephone number	A valid email address is needed for registration and to contact study participants (e.g., sending invitations to fill out questionnaires). A telephone number must be provided in case a participant cannot be reached via email (e.g., for follow-up surveys)
5) Fluency in the German language	To ensure that participants will be able to understand the information provided
**Exclusion Criteria**	**Rationale**
1) Participation in other psycho-social or pharmacological treatments for the reduction/cessation of cannabis use	To avoid confounding treatment effects
2) Current, pharmacologically-treated psychiatric disease or any history of psychosis, schizophrenia, bipolar type I disorder, or significant current suicidal thoughts	To avoid having subjects with these problems enter the study, and prevent confounding from other treatments

#### Assessments, compensation, and randomization

Once they complete a baseline questionnaire (t0), participants will be randomly allocated by a random number generated by the server to one of three study arms. The masking technique will be partially single-blinded: participants will know if they were assigned to the waiting list control group or to one of the two intervention groups. However, in the latter case, they will be unaware of the particular intervention that they are receiving. The risk of disappointing participants who fail to receive their preferred intervention is essentially eliminated by describing both interventions as ‘an optimized version of a previous program’ without providing further details. Those participants who are assigned to study arms 1 or 2 can then immediately commence with the program, whereas controls assigned to study arm 3 will be informed that they must wait for six months to access the intervention. Besides the baseline questionnaire (t0), participants in all study arms will be asked to fill out an online assessment at six weeks (i.e., for study arms 1 and 2: immediately after they conclude the program; t1), three months (t2) and 6 months (t3) after their baseline assessment. Participants will earn 20 Swiss Francs for completing the 3-month follow-up assessment (t2) and 30 Swiss Francs for completing the 6-month follow-up assessment (t3). They can individually decide whether they wish to receive the total amount as an online voucher or to donate it to a charity. The study personnel is not blinded.

### Interventions

The current section describes the contents of the active study arms. First, features that pertain to both study arms will be described. Second, the first study arm (mindfulness-based self-help IMI) will be described in detail, since the integration of mindfulness into the program is the primary novelty of CANreduce 3.0. Since study arm 2 (CBT-based self-help IMI) largely corresponds to the CANreduce 2.0 version [19], only major adaptions that have been integrated into the current version will be described.

#### Features of both active study arms (Study arms 1 and 2)

##### General set-up

Both study arms consist of eight modules that must be completed within a time frame of six weeks. The first four modules must be completed consecutively, after which participants might choose the order in which they complete the four remaining modules.

##### Tutorial

Participants familiarize themselves with the main features of the program via an initial tutorial that explains the dashboard, the consumption diary, the modules (including ‘my contributions’), the eCoaches, and the fictional companions (see subsequent paragraphs).

##### Dashboard

The dashboard displays useful information at a glance, including information on when the participant started the program and how many days remain for them to complete it. An overview of the different assessment time points and their status (completed, pending) is provided, as well. Similarly, an overview of the modules is given, including their title, their status of completion (completed, pending), and materials that the participant has created within the modules (e.g., personal lists of pros and cons of using cannabis). By clicking on the title of a module, participants are taken to that page in the module where they left off the last time they logged in. Participants also can enter their cannabis consumption over the past week via the dashboard, which is then displayed in the progress chart (see ‘consumption diary’ for details). The dashboard also includes an activity planner that asks participants to list one or more activities they have planned for the upcoming week, to indicate how much joy they are expecting from this/these activity/activities and whether they have already concluded it/them. This feature has been included because frequent cannabis use and depression often co-occur [55] and since activity enhancement is a key feature of CBT-based approaches to treat depression [23]. Similar components also are incorporated in the MBRP-program, wherein participants are asked to choose and engage in rewarding activities as a home practice [31]. Lastly, the eCoach accompanies the participant through the program via a message box, giving tips on what to do next (e.g., continue a module, fill out a survey, etc.).

##### Consumption (and mindfulness practices) diary

The consumption diary consists of two sections. In the first section, participants can fill out their personally-targeted consumption for the upcoming week by entering how many ‘standard joints’ they are planning to smoke each day (not smoking at all is indicated by entering ‘0’). The designation ‘standard joint’ refers to the information that the participant has provided in the baseline assessment (t0; see ‘Measurements’). In the second section, participants are asked to fill out how many standard joints they have actually smoked over the past week. The information they enter is then displayed in the progress chart, which compares planned with actual consumption within each particular week. Participants are encouraged to fill out their consumption diary at least once every week. Doing so, participants should gain a more detailed understanding about how many joints they are actually smoking. Furthermore, self-control over their consumption should be facilitated. Success (e.g., reduced cannabis use, in accordance with their personally set goals) can be visualized, as well. In study arm 1, any of the nine mindfulness practices that participants learn during the program can also be entered in this dairy by adding specific icons representing each practice. This might, for instance, allow participants to recognize a potential association between practicing mindfulness and their cannabis use.

##### eCoaches

For the tutorial offered before both active study arms, participants can choose either a male (Martin) or female (Anna) eCoach, who is depicted as an avatar. The selected eCoach accompanies participants throughout the program, by introducing each module and – at the end of it – summarizing the main contents and what they should have learned in written form. These text messages are identical with the male and female version of the eCoach. In study arm 1 (including mindfulness), the eCoaches also introduce themselves via an audio message that includes a semi-fictional background story on how mindfulness became an important part of their lives (the male and female introduction vary slightly). Adding these introductory audios in study arm 1 is important, as they allow participants to select the voice that sounds more sympathetic to them and is, therefore, preferred to guide them through the mindfulness practices of subsequent modules. The initially-selected eCoach is subsequently displayed by default in all modules, but can be changed at any time.

The eCoach will automatically send an email after individual’s register in the study and again at the end of each of the six weeks that participants are working on the program, whether they are in intervention arm 1 or 2. Those weekly emails are a way for the eCoaches to stay in touch with participants and accompany them through the course. They involve informing participants what week of the program they are in, suggesting certain modules, and reminding them to fill out the consumption diary. Additionally, participants who do not seem to use the program after registration or are lagging behind schedule are sent emails addressing the issue of concern and asked if they need any help. To automate this process, we predefined emails with specific trigger conditions like a) an empty diary in week 2, b) being in week 3 and having completed only one module, and c) reaching the end of the six weeks but having completed fewer than four modules. Adherence and retention should be improved through this emails.

##### Fictional companions

In the tutorial, participants can choose one of six fictional companions. Each has been given a name, age, profession, profile text, and avatar picture. Over the course of the program, the companions’ thoughts and experiences are displayed in written text at critical points within the modules, with the chosen companion displayed by default. The intention of this is to promote participants’ deeper reflection on certain key issues raised by the modules. Even though participants select a particular companion, they can toggle through the statements of the other fictional characters to gain insights into different perspectives. This program feature was already incorporated into CANreduce 2.0 but has been adapted for the current version. It manifests very similarly in the two active intervention arms, but occasionally differs between the two study arms, when mindfulness plays a role.

##### Other common elements

At any time during the program, participants can access further content, including information on relevant topics (e.g., on the immediate, medium-, and long-term effects of using cannabis) and on how to respond when immediate or additional help is needed, including a list of emergency numbers. In study arm 1, additional information is provided that includes an overview of all the mindfulness practices that participants will be taught in the different modules (including all audio files), information on the challenges that might be experienced practicing mindfulness, and further resources on mindfulness (videos, books, meditation-apps, mediation courses).

#### Study arm 1: mindfulness-based self-help IMI

For this study arm, the contents of CANreduce 2.0 have been adapted (see Section ‘Study arm 2: CBT-based web self-help) and new mindfulness content has been added with the aim of increasing the effectiveness of the previous version of the program, which was mainly CBT-based. Hereby, we were mainly drawing on the MBRP program [31], which combines mindfulness practices and cognitive and behavioral-based relapse prevention. Even though this program is typically delivered in a group format, Bowen et al. [31] suggested that it might also be useful to develop and test a web-based adaption to increase dissemination and access. Maximum flexibility regarding the starting point and timing of module processing, as well as user anonymity are key features of CANreduce. Therefore, we refrained from setting up an online group where participants meet virtually. Rather, we aimed to stimulate deepened reflection into what participants are experiencing during the mindfulness practices via displayed dialogs between the eCoach and the fictional companions (e.g., on intrusive thoughts during meditation), as well as through statements from the eCoach or companions within the modules. These dialogues and statements are aimed to mimic the *‘inquiry’* of the classical MBRP program, which involves guided discussion about the direct experiences of participants during and following mindfulness practices [31]. The mindfulness practices of CANreduce 3.0 are guided by the female or male eCoach and are available as audio files. The practices are introduced in written form, whereby it is also described how they might support participants in their attempts to reduce their cannabis use. Some of the core mindfulness practices – mindfulness breathing, body scanning, and SOBER breathing space (see below) – are available in different lengths, so participants can choose what is best suited to them (e.g., choosing a shorter practice on days when their personal schedule is particularly busy). Besides the clinician’s guide to MBRP, other mindfulness-based resources were considered, as well, when the contents of the modules were developed [32, 56, 57]. Table 2 provides an overview of the contents of the modules that are now described in more detail.

**Table 2:** Overview of contents and therapeutic approaches in the modules of study arm 1 (mindfulness-CBT-based web self-help

**Module**	**Content and therapeutic approaches / resources**	**Mindfulness practices** (adopted or adapted from the indicated source)
Module 1: introduction	-Pros and cons of using cannabis, consumption goal, confidence of change (MI techniques [14]) -Introduction into mindfulness [27, 31, 56, 57]	-Mindfulness breathing [58] -Body scan [58]
Module 2: trigger	-Reflecting on practicing mindfulness (including its challenges; (MBRP; [31]) -Identifying personal triggers and recognizing seemingly irrelevant but triggering decisions (CBT approach to relapse prevention; [25]) -Dealing with urges (MBRP; [31])	-Urge surfing [31] -Mountain meditation [31]
Module 3: craving	-Introduction to and detailed discussion of the concept of craving (MBRP; [31], CBT [59]) -Ways of dealing with craving: ‘SOBER breathing space’ (MBRP; [31])	- SOBER breathing space [31]
Module 4: (re)lapse	-(Re)lapse cycle with a particular focus on thoughts (MBRP; [31]) -Ways of breaking the relapse cycle (MBRP; [31]) -Dealing and coping with (re)lapses (including being self-compassioned) (MBRP; [31]) -Mindfulness in everyday life	-Self-compassion [56] -SOBER breathing space (with a focus on thoughts; [31]) -Walking meditation [60]
Module 5: time for yourself	-Dealing with stress [32] -Developing healthy sleep habits [61, 62] -Decreasing excessive ruminations [61, 63–67] -Strengthening social contacts [23]	-Recognizing thoughts as thoughts [56] -Lovingkindness meditation [56]
Module 6: addressing problems	-Relationships between consumption, problems, and low moods (depressive symptoms) [24] -Skills to deal with solvable and unsolvable problems, including cognitive [61] and mindfulness approaches (MBRP; [31])	In order to accept negative feelings that might arise from unsolvable problems, two practices are suggested – urge surfing and lovingkindness meditation (Links to these meditations are provided)
Module 7: lifestyle-balance, self-care and saying ‘no’ (refusal skills)	-Awareness of and nurturing lifestyle balance and self-care (MBRP; [31]) -Strengthening refusal skills for use in high-risk situations (based on CBT; [15])	-SOBER breathing space (with a focus on thoughts; [31]) -Lovingkindness meditation [56]
Module 8: preserving achievements	-Review of program -Writing down personal tips to help secure achievements after the program is complete (based on MI techniques; [14] -Emphasizing the importance of the continuation of practicing mindfulness (MBRP; [31]) -Overview of introduced mindfulness practices within the program -Further resources on mindfulness (books, videos, courses, etc.)	Link to an overview that includes all practices that were introduced within CANreduce 3.0

##### Module 1: introduction

In the first part of this module (mostly based on MI techniques [14]), participants are asked to consider all the pros and cons of using cannabis. A change in behavior is facilitated when the cons of using cannabis are perceived to outweigh the pros. However, even if the pros seem to outnumber the cons, participants may still be motivated to reduce their consumption; e.g., by affording some cons greater weight than others and by replacing ostensible pros with alternative behaviors. Participants are also asked to define their consumption goal and to indicate how confident they are that they can change their consumption. Advice on how to increase this confidence is given as well.

Mindfulness is introduced in the second part of the module (both in written form and in the form of an animated video), as is how it might help participants to reduce their cannabis use [27, 31, 56, 57]. Two formal mindfulness practices are introduced, and the importance of practicing mindfulness regularly is emphasized. Lastly, participants are encouraged to continue attending the program even if they feel that mindfulness is not for them.

##### Module 2: triggers

At the beginning of this module, participants are asked to reflect on their experiences with the mindfulness practices that were introduced in the first module. This reflection is intended to be stimulated through displayed dialogue between several companions and the eCoach (including a discussion of common challenges of practicing mindfulness, like intrusive thoughts [31]). Subsequently, its intention is to increase participants’ awareness of their personal triggers to use cannabis [25]. The mindfulness practice ‘urge surfing’ [31] is introduced next, as it might help participants to resist urges to use cannabis, even when confronted with a personally-relevant trigger. Subsequently, seemingly-irrelevant decisions and chains of events that can potentially lead to the use of cannabis are discussed [25]. Participants are given the opportunity to practice ‘mountain meditation’ [31] at the end of this module to strengthen their own foundation and gain new stability, which might be particularly important after they have focused on personal triggers.

##### Module 3: craving

The concept of craving and how it is connected to triggers (see previous module) and conditioned stimuli is introduced [31, 59]. Participants are asked to think about the ways in which they experience craving (i.e., the sensations, emotions, and thoughts that go along with it). It is also explained how craving might change in the short- and long-term, when a person decides to give in to the urge to use cannabis versus resist it. Lastly, participants are introduced to the ‘SOBER breathing space’ [31] that builds on the mindfulness practices of ‘mindfulness breathing’ and ‘body scanning’ introduced in earlier modules. The acronym SOBER represents the following five steps a person can go through to cope with challenges, stressful situations, and triggers that might evoke craving: 1) ‘Stop or slow down’ to avoid their usual automatic pilot response and bring awareness to the current moment; 2) ‘Observe’ what is happening (bodily sensations, emotions, thoughts); 3)‘Breathe’; 4) ‘Expand’ awareness to one’s whole body and the current situation; and 5) ‘Respond’ with awareness.

##### Module 4: (Re)lapse

The beginning of this module emphasizes how an initial lapse need not be a catastrophic event that inevitably leads to a full-blown relapse. Rather, participants are encouraged to learn from such lapses (e.g., since they might provide clues on issues that still require clarification) and, thereby, come to a clearer understanding of the path they ultimately must take to reduce their cannabis use as a long-term endeavor and learn to be patient and self-forgiving during this process. A core element of this module is its discussion of the relapse cycle: the chain of events that might lead to a (re)lapse (adapted from [31]). Hereby, the important role that thoughts play at each point in the cycle is emphasized [31] and participants are encouraged to think of personal examples. Furthermore, they are taught that slowing down and observing one’s own thoughts, emotional states, and physical reactions might create an opportunity for them to step out of the relapse cycle and their prior automatic pilot mode and arrive at a more considered response [31]. In this respect, participants are invited to use the SOBER breathing space (this time around with a particular focus on thoughts) [31].

The module also shows participants ways for them to cope with (re)lapses. Among other things, it emphasizes the importance of being self-forgiving in such situations and introduces corresponding mindfulness practices [56]. At the end of the module, the discussion’s scope is broadened again by explaining how mindfulness can be integrated into one’s everyday life and by introducing the concept of ‘walking meditation’ [60].

##### Module 5: time for yourself

In this module, participants are encouraged to take the time they need to work on their needs so they can improve their general well-being and, thereby, construct a solid foundation upon which they may reduce their cannabis consumption. How these needs might be impacted by the use of cannabis and how mindfulness (including specific mindfulness practices) might be helpful when working on these needs are illustrated. More specifically, participants learn how to (1) improve the way they deal with stress (e.g., by first stopping and becoming aware of what happens in stressful situations, so they can learn to respond in a more considered and less automatic manner) [32], (2) develop healthier sleep habits (e.g., by unwinding before getting into bed) [61, 62], (3) reduce ruminations ([61, 63–67], including the mindfulness practices ‘recognizing thoughts as thoughts’ [56] and ‘loving-kindness meditation’ [56]), and (4) strengthen their social contacts ([23]; e.g., by becoming aware of possible negative beliefs that might inhibit them from getting closer to people (the loving-kindness meditation [56] is suggested again for this purpose).

##### Module 6: addressing problems

The sixth module begins with a description of how low moods, problems, and the use of cannabis are connected and how this might ultimately create a vicious cycle [24]. Subsequently, participants learn how they can deal with both solvable and unsolvable problems. The following six-step plan is introduced to approach solvable problems: (1) define the problem; (2) define the goal that is aimed to be achieved; (3) search for possible solutions; (4) specify (small) steps to solve the problem; (5) try out possible solutions, and (6) take stock. Different techniques that might be useful for coping with unsolvable problems also are introduced (e.g., confiding in someone else or seeking contact with people with similar problems). It is also detailed how mindfulness might be of help for both solvable and unsolvable problems. Among other things, this module emphasizes that accepting ones’ feelings (including negative ones) is important. Participants also are encouraged to think about and work on their own solvable and unsolvable problems in the module.

##### Module 7: lifestyle-balance, self-care, and saying ‘no’ (refusal skills)

This module details how participants can nurture their lifestyle-balance and self-care to better achieve and maintain their consumption goal. Regarding their lifestyle-balance, participants are first asked to classify their daily activities into depleting (e.g., discouraging, exhausting, frustrating) and nourishing ones (e.g., energizing, pleasurable, satisfying; adapted from: [31]). Participants are then encouraged to plan some nourishing activities for the upcoming week and – if possible – to actively work on depleting activities (employing what they have learned in Module 6). However, it also is emphasized that this practice is not intended to eradicate all depleting activities in their life; but rather for the individual to become more aware about how their days are spent and how they might relate to certain experiences differently. Regarding self-care, participants are encouraged to develop a friendly, accepting, and benevolent attitude towards themselves and to practice the loving-kindness meditation [56]. The module also strengthens participants’ refusal skills and, by doing so, helps them to communicate clearer, more self-confidently, and less ambiguously that they no longer want to use cannabis (or not use it as often and/or as much) [15]. In this way, their risk of relapse when confronted with high-risk situations is reduced. The SOBER breathing space [31] is recommended again as a useful practice when they are confronted with high-risk situations.

##### Module 8: preserving achievements

In this final module, participants are asked to look back on the program, to visualize difficult and helpful moments, and to write down personal tips for maintaining their consumption goal. This is to prepare them for life after the program. At the end of the module, the eCoach also will point out how mindfulness was a core element of all the modules and will encourage them to continue practicing it regularly. Besides providing an overview of all mindfulness practices that were introduced in the previous modules, a list with further resources on mindfulness is provided (e.g., books, videos, courses).

#### Study arm 2: CBT-based self-help IMI

The main changes to study arm 2 in CANreduce 3.0, relative to CANreduce 2.0, can be summarized as follows. First, a tutorial has been introduced that familiarizes participants with the features of the program (see above) to increase the program’s usability. These contents have already been described in Module 1. Secondly, the order of the modules has been changed, and the first four modules must be worked through one after the other before any of the remaining four modules can be started. In contrast, users of CANreduce 2.0 were permitted to work on the modules in whatever order they chose. These changes, as well as some slight changes in the wording of the modules’ titles, have been implemented so study arms 1 and 2 in the current CANreduce 3.0 study are similar (note that the sequential order of the first four modules in the mindfulness-based study arm is necessary, as the concepts introduced in modules 1–4 build upon each other). Thirdly, some minor language and content improvements have been implemented in each module (e.g., we changed the personal pronoun from the formal "you" (German "Sie") to the informal "you" (German "Du")). Fourthly, mindfulness-related content that existed in CANreduce 2.0 has been deleted to avoid any confounding effects between the two active study arms of the current study. Lastly, the eCoach was adapted. Even though the previous study on the effectiveness of CANreduce 2.0 revealed that the study arm having an anonymous support team (and not the personal eCoach who introduced the modules via a video) was the most effective, we still used an eCoach rather than an eTeam in this study arm of CANreduce 3.0. However, since it is possible that the eCoach in CANreduce 2.0 did not match all the participants’ expectations (e.g., in terms of gender or age), the eCoach for CANreduce 3.0 is now displayed schematically (as an avatar picture) and participants can choose between either a male or female version. Furthermore, and analogous to study arm 2, the eCoach now introduces each module in written form and summarizes the main lessons learned at the end of each module.

### Technical specifications of CANreduce 3.0

CANreduce is a website accessed by an internet browser from a computer, tablet, or smartphone. The design automatically adapts to each device’s screen size (responsive design). While CANreduce 2.0 was based on Drupal 7, CANreduce 3.0 is based on Drupal 9. Drupal is an open source content management system that runs on PHP and MySQL/MariaDB. The website, including all data, runs on a litespeed webserver hosted by a professional company, and is located in a high-security data center in Switzerland. Physical access to this data center is only granted after biometric identification. All access via a browser is enforced through SSL encryptions. Participants have access to their account via their own username and password. The developers at the Principal Investigator’s (PI) institution have full access to all data from the participants (except their passwords) via personal administrator accounts. Data will be extracted at several time points (including a review of data quality) and stored at the PI's institution on local computers and local fileservers for analysis and archiving.

Participants can create an account by providing a username and e-mail-address. After clicking an e-mail verification link to ensure that the e-mail address they have entered is correct, they can create their password and start the baseline assessment procedure. All measurements in this study are entered via the website. Automatic input validation ensures a certain level of data quality. Sensitive data, like email addresses and phone numbers, will be deleted upon study completion.

### Safety

Potential risks to participants are assumed to be minimal. First, no drugs will be administered during the trial. Secondly, some people who might be at higher risk for adverse consequences are ineligible for the study (e.g., people with significant current suicidal thoughts). Thirdly, an instant help page will be available at all times that includes instructions on what participants might do if their life situation becomes unbearable (including emergency contacts). This instant help is mentioned explicitly at points in the program that might be particularly challenging for participants. It is expected, for example, that participants will experience some mild withdrawal symptoms — such as craving, mild depressive states, and sleep problems. These symptoms are mitigated by being explicitly addressed within the modules.

### Measures

Table 3  provides an overview of all measurements and when in the study they are used. All measurements are self-reported, assessed online, and detailed subsequently:

1. *Sociodemographic data* include age, gender, sexual orientation, educational attainment, financial situation, and migration background.
2. Problematic cannabis use is assessed with the revised version of the *Cannabis Use Disorders Identification Test (CUDIT-R* [68]*;)*. Each of the eight items contains a statement about cannabis use that must be answered on a 5-point Likert scale that ranges from "never" (0) to "daily or almost daily" (4). The total score ranges from 0 to 32 with scores of 8 or more indicating hazardous cannabis use and scores of 12 and more indicating a possible CUD.
3. Various further aspects of cannabis use are assessed. Regarding *consumption patterns: (3a)* the i) number of years using cannabis, ii) means of consumption (oral, smoking or vaporizing), and iii) number of days using CBD over the last 30 days are measured. Participants then *define their personal standard joint (3b)* by selecting between i) indoor, outdoor, and resin, ii) mixed with or without tobacco, and iii) six different quantities of the substance, varying between 67 and 500 mg. Each of the resulting 36 combinations is illustrated with a photo of an unrolled joint paper on which the specific amount of cannabis (and tobacco) that has been selected by the participant is displayed. A ruler beside the unrolled joint serves as a reference. This standard joint is used as a quantitative unit to assess 3c) the frequency and quantity of cannabis use in the last seven days, in accordance with the *Time-Line-Follow-Back (TLFB)* method [69, 70].
4. The *Severity of Dependence Scale (SDS)* is a five-item scale that can be used to screen for dependence. Each item must be answered on a 5-point Likert scale that ranges from 0 to 4. Total scores range from 0 to 20, with a score of 4 or more indicating cannabis dependence [71].
5. The *short version of the Alcohol Use Disorders Identification Test (AUDIT-C* [72]*;)* is a screening tool with three items that assess problematic and risky alcohol consumption. All items must be answered on a 5-point-Likert scale, that ranges from 0 to 4. The total score ranges from 0 to 12, with scores of 3 (women) and 4 (men) indicating problematic use and scores of 4 (women) and 5 (men) or more indicating risky use.
6. The *NIDA ASSIST* is an *Alcohol, Smoking and Substance Involvement Screening Test (ASSIST) from the National Institute on Drug Abuse (NIDA).* We only use the pre-screener [73] without the items on alcohol and tobacco. For each drug, consumption is rated, ranging from "never" (0) to "daily or almost daily" (4).
7. Drug Abuse Screening Test (DAST-10) is an instrument with ten yes–no questions about drug abuse [74]. Each answer is allocated either 0 or 1 point, leading to a total score between 0 and 10. Scores are interpreted as follows: 0 for no problem, 1–2 for a low level, 3–5 for a moderate level, 6–8 for a substantial level, and 9–10 for a severe level of abuse.
8. The *Perceived Stress Scale (PSS-10)* is an instrument with 10 statements about experiences of unpredictable, uncontrollable, and overwhelming life events. Answers range from "never" (0) to "very often" (4), leading to a total score between 0 to 40 with higher numbers indicating more stress, but without distinct any cut-offs for diagnostic purposes [75, 76].
9. The *Patient Health Questionnaire for Depression (PHQ-9)* consists of nine items that assess degree of depression. Answers range from "not at all" (0) to "almost daily" (3), resulting in a total score between 0 and 27 [77]. A score of 10 or higher is considered indicative of a major depressive disorder [78].
10. *The Generalized Anxiety Disorder Screener (GAD-7)* is a screener with seven items on anxiety symptoms, with answers ranging from "not at all" (0) to "almost daily" (3) and total scores between 0 and 21; any score of 10 or higher is considered indicative of a generalized anxiety disorder [79].
11. The Adult ADHD Self-Report Scale (ASRS-V1.1) is a six-item questionnaire on attention deficit and hyperactivity, with answers ranging from "never (0) to "very often" (4), leading to a total score between 0 and 24 [80]. Total scores range from 0 to 24 with values of 14 and above indicating a positive screening result for ADHD [81].
12. *The Mindfulness Attention Awareness Scale (MAAS)* is a questionnaire with 15 statements about everyday experiences in mindfulness, with answers ranging from "almost always" (1) to "almost never" (6), and higher mean scores indicating greater mindfulness [82, 83].
13. *The Comprehensive Inventory of Mindfulness Experiences (CHIME)* is a 37-item questionnaire with answers ranging from "almost never" (1) to "almost always" (6), with higher mean scores representing more mindfulness. This relatively large instrument has eight subscales (inner awareness, outer awareness, acting with awareness, acceptance, decentering/non-reactivity, openness, relativity, and insight) and covers all aspects of mindfulness employed in current mindfulness scales [84].
14. *Previous experience with meditation* is assessed with two questions about frequency (never, once, sometimes, regular) and guidance (innately learned, self-taught from books, learned in a course, guided by an app).
15. *The Client Satisfaction Questionnaire for Internet Interventions (CSQ-I)* is specifically designed to assess client’s satisfaction with internet health interventions. It consists of eight items with answers ranging from "disagree" (1) to "agree" (4), leading to scores between 8 and 32; higher scores indicate higher levels of satisfaction [85].
16. *Negative effects* of internet interventions are assessed according to Rozental [86]. Participants are asked if they have experienced any negative side effect that they attribute to the program. If so, they are asked to describe it and rate the severity of any resulting impairment.
17. *Use of any services besides CANreduce*, like online counseling, drug counseling, general practitioner, psychologist, or psychiatrist. Participants are asked to respond ‘yes’ or ‘no’ to all of these services and to list any other unlisted services used.
18. *Adherence and Retention:* Adherence is operationalized as average completion rate spanning the eight modules, with module completion rate defined as the highest page number visited divided by the highest page number available in a given module. Retention is operationalized as the average completion rate of the weekly consumption diary over the six-week program.

**Table 3:** Overview of all measures and their measurement time points

	**Assessment instruments**	**Base-line (t**_**0**_**)**	**6 weeks (t**_**1**_**)**	**3 months follow-up (t**_**2**_**)**	**6 months follow-up (t**_**3**_**)**
1	Socio-demographics (age, gender, sexual orientation, educational attainment, migration background, financial situation)	X			
2	Cannabis Use Disorders Identification Test (CUDIT-R)	X	X	X	X
3	*Cannabis use*				
3a	Consumption Patterns (Years of Use, 30-day point prevalence of cannabis and CBD abstinence, Type of Consumption)	X	X	X	X
3b	Defining the personal standard joint	X			
3c	TLFB (Cannabis use frequency and amount for the last 7 days in standard joint units)	X	X	X	X
4	Severity of Dependence Scale (SDS)	X	X	X	X
5	Short version of the Alcohol Use Disorders Identification Test (AUDIT-C)	X		X	X
6	National Institute on Drug Abuse *Screening (NIDA ASSIST)*	X		X	X
7	Drug Abuse Screening Test (DAST-10)	X			
8	Perceived Stress Scale (PSS-10)	X	X	X	X
9	Patient Health Questionnaire for Depression (PHQ-9)	X		X	X
10	Generalized Anxiety Disorder Screener (GAD-7)	X		X	X
11	Adult ADHD Self-Report Scale (ASRS-V1.1)	X			
12	Mindfulness Attention Awareness Scale (MAAS)	X	X	X	X
13	Comprehensive Inventory of Mindfulness Experiences (CHIME)	X			
14	Previous experience in meditation (no official instrument)	X			
15	Client Satisfaction Questionnaire for Internet Interventions (CSQ-I)		X^a^		
16	Negative effects, according to Rozental et al. (2015)		X		
17	Use of any services besides CANreduce			X	X
18	Adherence and Retention^b^		X		

^a^ Only for study arms 1 and 2

^b^ Adherence and retention are constructs measured indirectly and not part of a questionnaire

The primary study outcome is the number of days of cannabis use over the preceding 30 days.

Secondary outcomes are quantity and frequency of cannabis use over the last seven days’; scores for the SDS, AUDIT-C, NIDA-ASSIST, PSS-10, PHQ-9, GAD-7, MAAS, and CSQ-I., and any negative effects attributed to the program.

### Hypotheses

The following detailed study hypotheses for the main outcome (i.e., reduction in the monthly days of cannabis use between baseline and the 3- and 6-month follow-up assessments) will be tested:

1. A mindfulness-based self-help IMI for the reduction of cannabis use (study arm 1) is more effective than being on a waiting list (control condition, study arm 3).
2. A CBT-based self-help IMI for the reduction of cannabis use (study arm 2) is more effective than being on a waiting list (control condition, study arm 3).
3. A mindfulness-based self-help IMI for the reduction of cannabis use (study arm 1) is at least as effective as a CBT-based self-help IMI (study arm 2).

### Data analyses

Data will be analyzed according to the ITT principle. Multiple imputation procedures, using the R package *mice* [87], will be used to address missing data. *mice* involves specifying a multivariate distribution for the missing data and drawing imputations from their conditional distributions by Markov chain Monte Carlo (MCMC) techniques. We intend to use 20 imputed data sets, as this has been deemed sufficient [87]. The imputation model will include all primary and secondary outcomes (see above). Adjunct variables, like sociodemographic data, will be included if they improve convergence within the imputation model.

Differences in primary and secondary continuous outcome variables between the two active study arms (1 and 2) at baseline and at both follow-up points will be tested using linear mixed models (LMM). The LMMs will be specified to model clusters and repeated measures by defining random effects for study condition and time (repeated measures). Appropriate distributions for non-normal continuous outcomes will be specified (e.g., negative binomial, zero-inflated). In addition to the ITT analyses, per-protocol analyses will be performed.

To investigate the exploratory research question of whether study arm 1 (mindfulness-based self-help IMI) results in a similar reduction in days of cannabis use as study arm 2 (CBT-based self-help IMI), a confidence interval approach will be used for the ES of the difference between these two study arms, using a two-sided 0.05 level of significance [54]. The equivalence margin is a priori set at d = 0.20, corresponding to the smallest value that would constitute a relevant effect [88]. The upper bound of the 95% CI for the ES will be compared against the predefined equivalence margin of d = 0.20 and must be below the margin to show equivalence.

Additional exploratory regression analyses will be conducted to determine whether baseline variables predict the frequency or quantity of cannabis use, severity of dependence (SDS), reduced psychiatric symptoms (PHQ-9, GAD-7), perceived stress (PSS-10) and mindfulness (MAAS) at 3 and/or 6 months follow-up, treatment retention, and adherence. For these analyses, we will use linear, multinomial, or binary regression models, depending on the scale used for the outcome measure. Treatment retention and client satisfaction will be compared between study arms one and two by Pearson chi-square analysis at week 6 (intervention end).

### Power analysis

Following best-practice procedures for LMM, power analysis was based on a Monte Carlo simulation. Based on pre-specified LMM model parameters, new values for the primary outcome were simulated and tested by z-tests for the comparison between study arms 1 and 3 (main hypothesis). This procedure was iterated 1000 times using simulated replicates. Power resulted from the number of significant effects identified across simulations (see [89]). The model parameters were derived from the CANreduce 2.0-RCT [19], due to its similar study design and measurements. We estimated a two-level Poisson generalized linear mixed model (GLMM) with random slopes and fixed effects for "measurement time", "study condition", and "condition*time". The CANreduce 2.0 model estimate yielded an effect of b = -0.069 (*p* = 0.07) for the interaction. This value was reduced to -0.06 for the power analyses to account for the smaller ES that have been established in meta-analyses [10]. The simulation indicated a power > 80% for *n* = 210 per study arm. Hence, a total sample of 630 frequent cannabis users will be targeted.

## Discussion

To the best of our knowledge, this is the first RCT to test whether a self-help IMI that incorporates mindfulness is effective at reducing problematic cannabis consumption in adults (broad age range) with frequent cannabis use. Important limitations of previous research are taken into account; for instance, by aiming for a large enough sample size that allows us to detect even small ES. Furthermore, study arm 1 of CANreduce 3.0, which combines CBT and mindfulness, seeks to leverage the established effectiveness of IMI [10–13] (including previous versions of CANreduce [17, 20]) as well as mindfulness-based programs [33, 38–45] for the treatment of SUD (or frequent cannabis use, more specifically). It is possible that adding mindfulness to CANreduce 3.0 is particularly relevant for some subgroups of the population with certain clinical (e.g., co-occurring SUD and depression; [46, 47]) or sociodemographic characteristics (e.g. [48]). Furthermore, the RCT outlined here will provide significant insights into the questions of whether MBI can effectively be delivered via technology-based platforms and, by doing so, overcome some of the implementation challenges of traditional MBI [30]. More generally speaking, CANreduce 3.0 – if proven effective reducing cannabis use and related problems (e.g., symptoms of mental disorders) – could be of particular significance, as it could help to reach frequent cannabis users in the general population who are reluctant to seek traditional forms of help [8, 9] or lack access to similar programs (e.g., mindfulness programs offered in situ).

Despite the potential advantages of CANreduce 3.0, the following limitations must be taken into account. First, and based upon previous research (e.g. [17]), relatively large drop-out rates are expected. Furthermore, it is possible that adherence to IMI is generally limited, due to the distant nature of such interventions [90]. However, according to the supportive-accountability model, [18] it is assumed that these issues have been somewhat ameliorated in CANreduce 3.0, via the addition of both a female and male eCoach. Secondly, it must be acknowledged that many contents in the modules offered in study arms 1 and 2 are provided in written form. Hence, a certain level of literacy is required. Thirdly, all measurements will be self-reported; and, though most of the measurements used have been validated in other clinical and research settings, they have not yet been validated as online assessments. On the other hand, participants in studies assessing previous versions of CANreduce also filled out questionnaires online, which did not seem to cause any (validity) issues.

In conclusion, we believe that version 3.0 of CANreduce will further our understanding of the effectiveness of IMI and MBI (and their combination) for the treatment of frequent cannabis users and will – if found effective – help to reach and support frequent cannabis users who might otherwise forgo care.

## Data Availability

Not applicable.

## Abbreviations

CBT: Cognitive-behavioral therapy
CUD: Cannabis use disorder
CUDIT-R: Cannabis Use Disorders Identification Test-Revised
ES: Effect size
GLMM: Generalized linear mixed model
IMI: Internet- and mobile-based intervention
ITT: Intention-to-treat principle
LMM: Linear mixed models
MBI: Mindfulness-based intervention
MBRP: Mindfulness-based Relapse Prevention
MBSR: Mindfulness-Based Stress Reduction
MCMC: Markov chain Monte Carlo
MI: Motivational interviewing
MORE: Mindfulness-Oriented Recovery Enhancement
MTS: Mindfulness Training for Smokers
RCT: Randomized controlled trail
SDS: Severity of Dependence Scale
SUD: Substance use disorder
TAU: Treatment as usual
TLFB: Time-Line-Follow-Back
THC: Tetrahydrocannabinol
